# Dentition and Its Derangements

**Published:** 1862-12

**Authors:** A. Jacobi


### Selections.

---

Dentition and its Derangements—*By A. Jacobi,* M. D. —*LECTURE X.*—Part 3.—The nervous system of children has hitherto been the subject of our consideration, inasmuch as the circulation of the blood is influenced by it. It is, however, but just that we now consider it in that light and also in relation to its other functions. This is not only an important and not very easy task; not very easy, as both of its anatomical structure and the physiological actions its morbid conditions frequently resist a successful exploration; very important, from the very frequency of cerebral and other nervous affections of a more or less severe nature. Mauthner asserts to have observed 1,747 cerebral diseases in 15,836 cases of sickness in children. According to Dr. Forsyth Meigs, there were in Philadelphia, in the course of five years, 3,970 deaths in children from diseases of the brain, 4,204 from diseases of the digestive organs, and 3,376 from diseases of the respiratory organs; and West remarks, that of 16,258 deaths resulting from diseases of the nervous system, in London (1842 and 1845), 87 per cent. were observed during the first five years. These are deaths. Only long-continued statistical reports on the occurrence of nervous diseases and functional troubles, not followed by fatal termination, could give a correct idea of the numerical prevalence of such affections. The number of deaths, however, is already so large, that as not only material organizations but also functional disorders of the brain, spine, and nerves, are frequently observed, the per centage of nervous affections, both fatal and not, is enormous. As to material changes of the substance of the nervous system, it is true that many diseases of adults are not, or rarely found in early age, for instance, carcinomatous and sarcomatous degeneration, certain forms of encephalitis, etc., but there are a number of prevalent forms by far more frequent in children than in more advanced age. Amongst these latter, I count anomalies of the amount of blood contained in the blood vessels of the nervous system, particularly its centres; meningitis in two forms, both genuine and tubercular; apoplexy; hydrocephalus; and also hypertrophy. Mere functional disorders, that is, such diseases of which with our limited means of investigation, and narrow knowledge, we have not been able hitherto to find the anatomical foundation, are also frequent. I need but allude to the frequency of convulsions in infantile age, which in a large per centage of cases will pass away without apparently leaving behind themselves any serious consequences.

I have stated that our knowledge of the more minute anatomy, and also the physiology of the nervous system, especially in its anomalies, is still very limited; nevertheless, those facts which anatomical researches have pointed out, will lead to the conclusion that the prevalence of nervous diseases in early age, is readily explained by the nature of the infantile nervous system. This is principally true as far as the brain and skull are concerned, as the spine and nerves, being less amenable, have been less closely studied. The anatomical facts to which, before entering into the discussion on the connection of dentition with nervous affections, I desire to direct your attention, belong principally to the condition of the cranial bones and the cerebral substance.

In the newly-born, ossification has made most progress in the auditory bones, labyrinth, portion of the petrous bone, and lower maxilla. The frontal bone still consists of two halves, its tuberosities are prominent, and no frontal cavity has commenced to be formed before the end of the first year. The occipital bone consists of four parts, the sphenoid of three; the parietal bones have not yet acquired their square condition, have as it were a fibrous appearance, and prominent tubera. The temporal bone is still divided into four parts (the petrous, mastoid, and squamous portion, and the annulus tympani); the ethmoid into three, with very few signs of ossification. There is still, in the upper maxilla, a sign of the presence of the intermaxillary bone; the maxilla containing the alveoli for two incisors, the canine, and two molar teeth, and a distinct but very small antrum Highmori. The palate bones are low, little developed, but consist already of a single piece. The inferior maxilla is divided in two halves, and contains the twelve central alveoli. In the upper and lower dental alveoli there are already the gelatinous germs of the twenty temporary and the first four pemanent teeth.

The cranial bones are kept in connection by sutures and sutural cartilage, this latter being the remnant of what has surrounded the cranial cavity in early fœtal life; the pericranium adhering more tightly to them than to the bones themselves. The large quadrangular fontanel, formed by the coronal and sagittal sutures, is large, of the size of a square inch and more, larger in large heads, smaller in small, and of enormous extension in hydrocephalic ones; the normal period of its closure being from the thirteenth to the fifteenth month. After this age it is found open in rachitical children; that persons in advanced life should have an open quadrangular fontanel, is an excessively rare occurrence. A few cases of this anomaly have been given in my essay on the premature closure of the cranial sutures and fontanels, and its etiological and prognostic importance, and an additional one has come under my notice a short time ago, in a young lad whose fontanel was still somewhat pulsating at the age of twelve years. The triangular small fontanel formed by the sagittal and lambdoidal sutures is closed in the mature child, as well as the lateral ones. Additional ones are rare, and then are only the results of ossification having commenced from a number of supernumerary points. The peculiar undulated shape of the sutures, or rather the margins of the cranial bones, is but gradually transformed into the dentated form, which is the common appearance later in life, after the third year. Real ossification will not commence before the twentieth year, and then first on the inner side of the cranium. Congenital or premature ossification of the sutures, or even simple closure, gives rise to serious consequences. Unilateral premature closure interferes with the symmetrical development of both skull and brain; if universal, it suppresses the normal growth of both. Microcephalus and idiotism result therefrom, or at least, from the brain being compressed, anomalies of the cerebral functions will be the consequence. Epilepsy or paralysis, naturally incurable, will follow. Or, in milder cases, the constant pressure on the cerebral substance will, by itself, or in the course of inflammatory or feverish diseases, bring on cerebral symptoms which, from the nature of the complication, will be seldom suppressed, and generally prove fatal. Perhaps the indistinct knowledge of this fact has induced old authors to call the Wormian bone, contained in a supernumerary fontanel of the frontal suture, from its apparently giving more room to the anterior portion of the brain, by the name of, not only interfrontal, but also anti-epileptic bone.

The head of the newly-born has an irregularly round shape; its height amounts to about the fourth part of the length, its weight to about a fifth of the weight of the whole body. Its principal diameters are these: Four or five inches from occipital to frontal, three and a half or three and three-quarters between the two parietal bones; five or five and a half from occipital bone to chin, three and a half from forehead to chin; its longest circumference is from fourteen to sixteen inches; its shortest, over the vertex, ten and a half to eleven inches. Its greatest width falls a little below the two parietal protuberances. The bones of the cranium are more injected with blood, bluish, and are more easily inflected than fractured; it predominates over the face. The frontal and parietal tuberosities are prominent. The upper margin of the parietal bone stretches in an almost perpendicular line above the lower one. The occipital bone lies more horizontally. The points of insertion of the muscles are less developed; nor are the superciliary arches very distinct; they are formed with the development of the frontal sinuses, whose first beginning dates from the second year, but which scarcely are worthy of the name before the tenth. The hair covering the scalp is short, thin, and often copious, the aponeurosis thin. In proportion to the general growth of the body the face gradually commences to predominate, the basis of the cranium growing fastest; mostly so in its posterior portion, together with the rapid growth of the occipital bone before the fifth year, while between the fifth and tenth year, in the period of the protrusion of the posterior molar teeth, the anterior portion develops at a more rapid rate. Gradually the frontal bone appears flatter, for several reasons; for its superior margin ascends; its inferior one is drawn anteriorly by the gradual prominence of the superior maxilla; and the formation of the frontal sinuses helps to bring on the same result. The occipital bone by and by loses its horizontal position, becoming more perpendicular.

After all, the several parts of the head do not grow at the same rate. The parietal bone has its full circumference at four years, while the frontal bone still continues growing. For this reason, the parietal portion of the large hemispheres of the brain is soon left behind by the anterior frontal lobe. The cranial cavity of the new-born is about a fourth or a third of that of adults, but already in the second year grows from 482 cubic centimetres up to 999, while the weight of the skull at that period reaches already three times its original amount. The occipital portion of the cranial cavity is very small in the newly-born, being only 5 per cent. of the whole. The frontal portion is as much as 13.89, the parietal 81.11 per cent. But as early as the second year, both the occipital and the frontal vertebræ grow each by 0.5 per cent. All these facts you will find to be in strict correspondence with the remarks I shall have to make on the relative development of the single portions of the brain.

The dura mater adheres tightly to the cranium, in infantile age, partly by means of blood-vessels, and partly by conglutination with the sutural cartilages. In the newly-born it is loosely attached opposite the parietal tuberosities. It is firmly adjacent to the cerebral surface, strongly injected, bluish and transparent. With advancing age it gains in solidity, but loses its transparency and injection. The arachnoid membrane and pia mater are very thin, colorless, transparent, and fragile; they show large veins filled with dark blood; there is a good deal of cerebro-spinal fluid, but no Pacchionian granulations are observed. The choroid plexus are of a similar nature to that of the meningial membranes; they are delicate, and contain more blood. All these facts constitute just as many differences from the condition in which the same parts are found in more advanced childhood, in adults, and in senile age.

The brain shows a lower degree of development. Its substance is less white, more transparent, and of a reddish-greyish color. It is of almost gelatinous consistency, at all events much less solid than in advanced age, and the division into medullary and cortical substances is less distinct. Only some parts excel by their proportionate hardness and their white color, viz.: medulla oblongata, corpora quadrigemina, corpora mammillaria, thalami, and pons Varolii. The fibrous appearance of the large hemispheres is not yet recognisable; the gyrations are thicker, less prominent, and fewer. The lateral ventricles exhibit less serous contents, and their walls are more even. The substance of the brain, finally, contains but little serous blood; only near the borders of the thalami there are a large number of blood specks.

Not only are these general differences between the brain of the infant and the adult recognisable in every specimen, but there are some which are of quite a specific anthropological importance. The relation of the several parts of the brain to each other changes according to age, as is well proven by accurate measurements, and weighing. The relative weight, for instance, of the large hemispheres, and the occipital portion of the brain, are particularly instructive. According to Huschke the cerebellum of the newly-born weighs 25 grammes, that of the adult from 180 to 193 grammes; that is, seven or eight times as much as the former. The large hemispheres of the newly-born weigh 300 grammes, those of the adult from 1,200 to 1,400, that is, four or five times as much as the former. The per centage drawn from the foregoing are these:—In the newly-born the cerebellum weighs 6 or 7 per cent., the large hemispheres 94 or 93, of the weight of the entire brain. Already after seven or twelve weeks these figures have been found to vary; the per centage of the cerebellum increasing to 9 or 11 per cent. At ten or fifteen years it has been found to be 12 or 13, nearly as much as in adults, where the per centage of the cerebellum is 12 or 14, of the large hemispheres 88 or 86.

A few other per centages are given by Huschke, viz.:—Male fœtus of five months, 5.14; female fœtus of seven months, 6.05; of eight months, 7.06; new-born female, 7.32; female of three years, 12.20; male of three years, 11.91; and female of fifteen years, 12.29. The figures of Chaussier differ somewhat: the proportion of cerebellum to the large hemispheres being from 1 : 13 to 1 : 30, or from 3.28 to 7.7 per cent. The same proportions, with all the astonishing differences, have been found to exist by Gall.

The forehead of the newly-born infant is narrow and low; the anterior lobes of the large hemispheres must consequently be expected to be proportionately small. Weighing and measuring confirm this conclusion. The anterior lobes in a prematurely born female, yielded only 16.5 per cent. of the weight of the entire large hemispheres; in the new-born, at full term, 22.09; in some who were from eight to twelve weeks old, also but from 16.7 to 18.9 per cent. This figure increases to 22.4 in the first twelvemonth. Whenever a similar figure, for instance 21.8 at seventy-seven years, occurs at advanced ages, circumstances must be peculiarly unfavorable. The remaining, especially the parietal portion of the large hemispheres, must necessarily show the reverse proportion. You remember the figures I gave for the frontal and parietal vertebræ. Now, the anterior lobes of the large hemispheres in the newly-born, weigh from 60 to 70 grammes; in the adult 300—that is five times as much as in the former; the remaining parietal and inter-parietal portion, however, in the newly-born 250, in the adult 1,000 grammes, that is four times as much as in the former.—*Am. Med. Times.*

